# Using a Family Systems Approach to Treat Tobacco Use among Cancer Patients

**DOI:** 10.3390/ijerph17062050

**Published:** 2020-03-19

**Authors:** Ellen Ruebush, Sara Mitra, Colleen Meyer, Laurel Sisler, Adam O. Goldstein

**Affiliations:** 1 Lineberger Comprehensive Cancer Center, The University of North Carolina at Chapel Hill, Chapel Hill, NC 27759, USA; saramitra@unc.edu (S.M.); adam_goldstein@med.unc.edu (A.O.G.); 2 Department of Family Medicine, The University of North Carolina at Chapel Hill, Chapel Hill, NC 27759, USA; colleen_meyer@med.unc.edu (C.M.); laurel_sisler@med.unc.edu (L.S.)

**Keywords:** tobacco treatment, cessation, family systems, family interventions, smoking cessation

## Abstract

Tobacco use treatment is an essential component of cancer care. Family members play a significant role in smoking behavior, but more research is needed regarding the development, implementation, and impact of family-based interventions in cancer care. The UNC Tobacco Treatment Program conducted an 18-month pilot study to examine the feasibility of implementing a family systems approach to treat tobacco use among patients at the North Carolina Cancer Hospital and to measure the impact of such an approach on patient abstinence. Implementation included four phases: (1) modifying the electronic health record and monthly report generated from the electronic health record; (2) training Tobacco Treatment Specialists to provide family counseling; (3) integrating family members into patients’ treatment; and (4) conducting six-month follow-up calls. During the course of the study, 42% (N = 221/532) of patients had family members integrated into their tobacco use treatment. Only 21 patients (4%) had family members present but not integrated into the treatment plan. At the six-month follow up time point, the seven-day point-prevalence quit rate for patients with family integration was 28% (N = 56/200), compared to 23% (N = 67/291) (*p* = 0.105) for patients without family integration. Integration of family members is clearly possible in an academic medical center’s oncology tobacco treatment program. Although pilot results were not statistically significant at 6 months, a potentially higher quit rate suggests a need for expanded research on methods to integrate family members in oncology settings for patients with tobacco-related cancers.

## 1. Introduction

Smoking is a risk factor for several types of cancer, including cancers of the head and neck, esophagus, lung, liver, kidney, pancreas, cervix, bladder, stomach, colon, rectum, and acute myeloid leukemia [1]. Studies show that continued smoking following a cancer diagnosis increases the risk of cancer recurrence, the risk of developing a new primary cancer, and rates of adverse treatment-related side effects [1,2]. Smoking cessation after a cancer diagnosis has been linked to a decreased risk of new cancers and longer survival [2]. Tobacco use treatment (TUT) is, therefore, a key component of cancer treatment, and efforts should be made to consistently improve interventions for cancer patients.

One such improvement could be the integration of family members and social supports into cancer patients’ TUT. A partner’s smoking status is a determining factor for smoking behavior change among adults [3,4,5], and family members play a significant role in smoking behavior, as evidenced by the correlation between family members’ behaviors—both supportive and unsupportive—and the likelihood that a smoker will make a quit attempt and achieve abstinence [6,7,8]. For example, family members continuing to smoke in the presence of lung cancer patients who had quit smoking caused patients worry and concern, exposed patients to secondhand smoke, and may have increased patients’ risk of relapse [9]. On the other hand, family members who pressured smoking cancer patients to quit smoking actually discouraged smoking cessation efforts among patients who wanted to maintain personal control over their behaviors in the context of a cancer diagnosis and treatment [10]. However, studies that have examined the effects of family-based versus individual-based interventions on smoking cessation outcomes among non-cancer patients have not found significant differences in quit rates [7,11,12], suggesting that more research is needed regarding the development and implementation of family-based interventions [13], particularly as part of routine cancer care.

In 2017, the National Cancer Institute (NCI) launched the Cancer Center Cessation Initiative (C3I) with the purpose of starting or expanding TUT programs at NCI-designated cancer centers [14]. With increased resources, C3I provided the UNC Tobacco Treatment Program (UNC TTP) with an opportunity to examine the implementation of a family systems approach in treating tobacco use among cancer patients. This study aims to test the feasibility of systematically integrating family members into patients’ TUT at the North Carolina Cancer Hospital (NCCH) and measure its impact on quit rates.

## 2. Materials and Methods

UNC TTP conducted an 18-month pilot study to determine the feasibility of implementing a family systems approach to TUT with patients of the NCCH. Implementation included four phases: (1) modifying the electronic health record (EHR) and monthly report generated from the EHR; (2) training Tobacco Treatment Specialists (TTS) to provide family counseling for substance use; (3) integrating family members into patients’ TUT; and (4) conducting six-month follow up calls to analyze quit rates. Feasibility was determined by the ability to train TTS in family counseling strategies, integrate family members into patients’ TUT, and document family integration. Two specific outcomes were measured: the number of patients who had family members integrated into their TUT and the difference in quit rates between these patients and those who did not have family members integrated.

**Figure 1:**
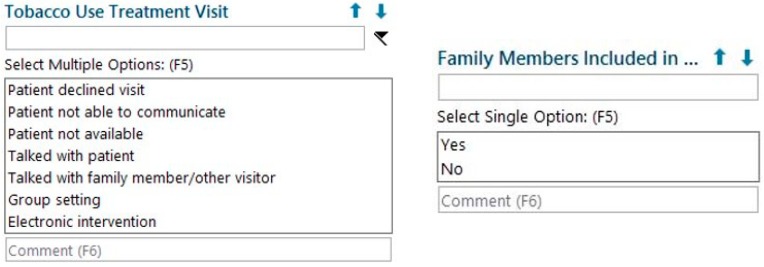
Fields added to the EHR documentation tool used by Tobacco Treatment Specialists (TTS).

UNC TTP modified the existing EHR documentation tool used by TTS as well as the monthly report pulled from the EHR by working with UNC Health’s Information Services Department. The existing documentation tool collected information about the patient’s current tobacco use, tobacco use history, and treatment plan. Two fields were added to this tool: (1) “Tobacco Use Treatment Visit”, which allowed TTS to document a family member’s presence in the session, and (2) “Family Members Included”, which allowed TTS to document whether that person was integrated into the patient’s TUT (Figure 1). The monthly report was modified to include these two fields, which enabled UNC TTP to monitor the status of the pilot and evaluate outcomes.

To prepare TTS to provide family counseling, UNC TTP invited a clinical professor from the UNC School of Social Work to provide a two-part, four-hour training on the topic of substance use counseling for families. In the first part of the training, TTS learned family counseling strategies and participated in role play exercises. These involved the following scenarios: family members who were difficult to engage, overly critical or overly involved family members, family members who also used tobacco, and family members who did not use tobacco. The second part of the training occurred two months after the first, and during this second training, TTS reviewed family counseling strategies, discussed specific cases and challenges encountered, and participated in additional role plays.

Following this training, TTS began providing family counseling to patients who had appointments at any one of the departments in the NCCH. Smoking patients were identified through the EHR and were referred by medical providers for TUT. Patients’ TUT—with or without family integration—was defined as at least one face-to-face session of assessment, behavioral counseling, and medication advice. TTS provided telephone follow-up TUT sessions if patients agreed and were available. On average, patients received 1.5 TUT sessions.

UNC TTP used an opt-out model for family counseling, so family members were integrated into patients’ TUT whenever they were present in patients’ sessions. Integration occurred by TTS briefly assessing family members’ tobacco use and desire to quit, making treatment recommendations to family members when appropriate, and identifying ways family members could help patients become tobacco-free. Integrated family members were typically spouses or partners, but were occasionally adult children, siblings, or parents of patients. Close friends present during patients’ sessions were treated as family members. TTS sought to integrate family members whether or not they used tobacco, believing that all social supports can provide assistance and encouragement to the patient irrespective of their own smoking status.

TTS contacted all patients at six months following their initial session to re-assess their tobacco use status. Intent-to-treat quit rates were calculated and based on self-reported seven-day point-prevalence abstinence. A one-tailed, chi-square test was used to determine if there were statistically significant differences in quit rates between patients who had family members integrated into at least one TUT session and patients who did not.

## 3. Results

**Figure 2:**
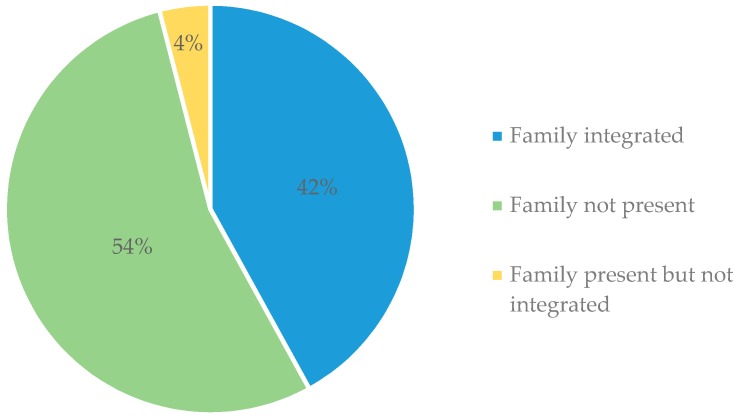
Family integration in patients’ tobacco use treatment (TUT), N = 532.

During the 18-month pilot study, 532 patients received TUT. Family members were integrated into TUT for 221 (42%) patients, and 21 patients (4%) had family members present but not integrated. Reasons for this included family members declining to participate in TUT and time constraints related to TTS borrowing clinic rooms that medical providers sometimes needed. TTS found no significant difference in time spent in individual counseling sessions, as compared to those that included family integration. Finally, 290 patients (54%) never had family members present during TUT (Figure 2).

The demographics of the 532 patients who received TUT are shown in Table 1. Most patients were White (69.2%) and African American (22.7%), and their age as expected was >45 (87.6%). No significant differences were seen due to family integration based on the demographic factors.

**Figure 3:**
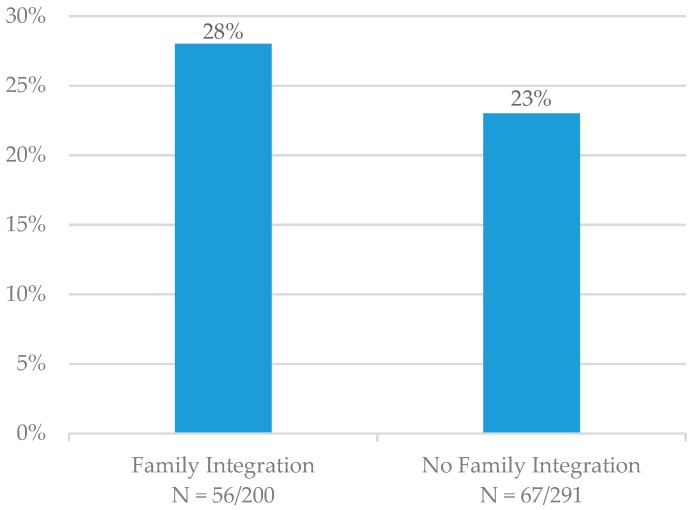
Six-month seven-day point-prevalence quit rates.

At six months, the seven-day point-prevalence quit rate was 28% (N = 56/200) for patients who had family members integrated into TUT and 23% (N = 67/291) for patients who did not (Figure 3). Patients who entered a hospice or were deceased at the six-month follow-up time point (N = 41) were not included in these calculations. The quit rate was not significantly higher for those patients who had family members integrated into TUT (*p* = 0.105).

## 4. Discussion

This pilot demonstrated that it is feasible to implement a family systems approach to TUT for cancer patients. UNC TTP made modifications to the EHR and to monthly EHR reports, which allowed the monitoring of the family systems approach and evaluation of outcomes. UNC TTP also successfully trained TTS in family counseling strategies, TTS were consistently able to integrate family members into patients’ TUT, and TUT sessions with family integration were not significantly longer than individual TUT sessions. During the 18-month pilot study, close to half of the patients who received TUT at the NCCH had family members integrated, and the primary barrier to family integration was the absence of family members in patients’ TUT sessions. Future studies could address this barrier by encouraging patients to bring a family member or social support to their counseling sessions. A secondary but consequential barrier that occasionally affected TUT with and without family integration was the fact that TTS, similar to staff in other ancillary services, often borrowed provider clinic rooms, meaning that some sessions had to be brief. Having an office dedicated to TUT would eliminate time constraints experienced by TTS.

While family integration occurred successfully, the six-month quit rate was higher but not statistically significant for those patients. Several reasons may exist for these findings. First, family member integration may not have been sufficiently intensive. In this study, family integration was defined as integration in at least one session of TUT, to try to make the intervention more generalizable to other settings, but more sessions with family integration may be required to achieve significant positive results. Second, this study did not account for differences in the relationship between the patient and the family member or differences in the family members’ tobacco use status. Prior research has focused primarily on the positive impact of partner or spousal support [3,4,5,6,7,8], and it is reasonable to believe that integration of a spouse or partner would have more effect than integration of a close friend, adult child, sibling, or parent. Similarly, it is plausible that integration of a family member who also smokes and is willing to quit could have a greater impact on patients’ abstinence than integration of a family member who does not smoke or is not willing to quit. Third, the monthly report pulled from the EHR did not collect information regarding cancer type or patients’ tobacco use history, and smoking cessation was not biochemically confirmed.

Finally, the pilot may not have had sufficient power to demonstrate the improvement. As a pilot, we did find a 22% improvement in quit rates, and with additional numbers of patients, these results could become statistically as well as clinically significant. Future research should expand upon this pilot by increasing both the number of patients with family integrated into TUT and the intensity of family integration. In addition, an examination of the differential impact of family member relationship and tobacco use status on patients’ abstinence would be valuable.

This is the first study to our knowledge reporting a low-cost, low-intensity quality improvement intervention to involve family members in the care of index cancer patients that smoke. A recent cohort study reported a very high intensity and expensive program in a cancer center, including 7–9 total counselling sessions, psychological or psychiatric intervention as needed, 10–12 weeks of free pharmacotherapy, and extensive staff-supported follow-up at 3, 6, and 9 months [15]. Their intention to treat nine-month self-report cessation rate was 35.6%, but the cohort also had exclusions. Our program achieved a 28% intent to treat self-report cessation rate with 1–2 counselling sessions with family involvement, no psychiatric treatment, and no free pharmacotherapy. It is difficult, however, to compare longitudinal cessation rates across studies without placebo or control groups. Additional interventions are needed to determine the optimal dose and strategies for sustainable interventions involving families that may improve outcomes.

## 5. Conclusions

An 18-month pilot study at the NCCH determined that implementation of a family systems approach in treating tobacco use among cancer patients is feasible if (1) family members are present in patients’ TUT, (2) TTS are adequately trained in family counseling, (3) modifications are made to the EHR, and (4) TUT programs commit to integrating family members into patients’ TUT. Although patients who had family members integrated into TUT had a higher quit rate than patients who did not, these results were not statistically significant. Future research should expand upon the pilot by increasing the number of patients treated, increasing the intensity of the family integration protocol, and evaluating specific characteristics of patients and family members.

## Figures and Tables

**Table 1:** Demographics of patients who received TUT.

	Received TUT (N = 532)	Family Integrated (N = 221)	Family Present, Not Integrated (N = 21)	Family Not Present (N = 290)
**Race, No. (%)**				
White	368 (69.2)	158 (71.5)	14 (66.7)	196 (67.6)
Black or African American	121 (22.7)	42 (19.0)	5 (23.8)	74 (25.6)
American Indian or Alaska Native	8 (1.5)	5 (2.3)	0 (0.0)	3 (1.0)
Asian	2 (0.4)	1 (0.4)	0 (0.0)	1 (0.3)
Unknown	33 (6.2)	15 (6.8)	2 (9.5)	16 (5.5)
**Sex, No. (%)**				
Male	294 (55.3)	140 (63.3)	9 (42.9)	145 (50.0)
Female	238 (44.7)	81 (36.7)	12 (57.1)	145 (50.0)
**Age, No. (%)**				
18–24	3 (0.6)	3 (1.3)	0 (0.0)	0 (0.0)
25–44	63 (11.8)	23 (10.4)	5 (23.8)	35 (12.1)
45–64	327 (61.5)	129 (58.4)	13 (61.9)	185 (63.8)
65 and older	139 (26.1)	66 (29.9)	3 (14.3)	70 (24.1)
